# Alogliptin Enhances Implant Osseointegration in Diabetes Through an Osteogenic–Angiogenic Immunomodulatory Procedure

**DOI:** 10.3390/ijms27156800

**Published:** 2026-07-29

**Authors:** Xiangdong Liu, Zijun Chen, Liu Yang, Wei Ma, Yiwen Liu, Yingliang Song, Mingchao Ding, Lei Tian, Mingkai Li

**Affiliations:** 1 Department of Pharmacology, School of Pharmacy, Air Force Medical University, Xi’an 710032, China; liuxiangdong@fmmu.edu.cn (X.L.); yiwen@fmmu.edu.cn (Y.L.); 2 State Key Laboratory of Oral & Maxillofacial Reconstruction and Regeneration, National Clinical Research Center for Oral Diseases, Shaanxi Clinical Research Center for Oral Diseases, Department of Oral and Maxillofacial Surgery, School of Stomatology, The Fourth Military Medical University, Xi’an 710032, China; dingmingchao@fmmu.edu.cn; 3 State Key Laboratory of Military Stomatology and National Clinical Research Center for Oral Diseases and Shaanxi Engineering Research Center for Dental Materials and Advanced Manufacture, Department of Oral Implants, School of Stomatology, Air Force Medical University, Xi’an 710032, China; dimi@hku.hk (Z.C.); lumos@fmmu.edu.cn (L.Y.); mawei@fmmu.edu.cn (W.M.); songyingliang@fmmu.edu.cn (Y.S.)

**Keywords:** diabetes, osteointegration, alogliptin, hydrogel

## Abstract

People with diabetes often have a high rate of oral implant failure and unstable long-term treatment effects. It is urgent to find the potential mechanisms and effective cure methods to address the increasing demand for dental restoration in recent years. This paper aims to find an influential factor, and a probable treatment resulting from it, to improve poor osseointegration and achieve favorable bone regeneration. To find out the influential factor of poor diabetic osseointegration, we compared marginal bone loss among patients taking different hypoglycemic drugs. To verify our pre-clinical findings, we performed in vitro experiments as well as micro-CT, fluorescence intensity, and histopathological analysis methods after establishing a diabetic rat model controlled by different systemic hypoglycemic drugs. To enhance implant osseointegration and find out the underlying mechanism, we proposed the GelMA–alogliptin hydrogel system and tested the scratch wound healing, cell adhesion, CCK-8, live-dead cell staining, osteogenesis, qPCR, and Western blot methods based on diabetic rat-derived BMSCs, HUVECs and BMMS, as well as the in vivo experiments in diabetic rats. People with diabetes under DPP4i (alogliptin) exhibit the lowest marginal bone loss. The systemic alogliptin and locally delivered peri-implant GelMA–alogliptin hydrogel treatment promoted bone regeneration around diabetic implants, enhanced diabetic-derived BMSC activity and migration through GLP1R/GSK3β/β-catenin and induced osteogenic differentiation, and promoted vascular regeneration and inflammation control. Alogliptin has positive effects on the oral implant health of people with diabetes. A locally delivered GelMA–alogliptin hydrogel system exhibits competitive ability to improve diabetic bone microenvironment and enhance implant osseointegration.

## 1. Introduction

Oral implants are the most common method for restoring tooth loss in individuals with dental defects or deficiencies. Their success rate has improved with advancements in surgical techniques and enhancements to implant surfaces. However, many systemic diseases impact their success [1,2].

Diabetes mellitus (DM) is a metabolic disease characterized by hyperglycemia due to defective insulin production, secretion, or both [3]. DM is mainly divided into two types, among which type II diabetes, which is characterized by the resistance of tissues to insulin, accounts for over 95%. Approximately 463 million people worldwide lived with type II diabetes mellitus (T2DM) in 2019 [4]. The number of estimated T2DM cases has risen by 62% over the decade [5]. Due to persistent hyperglycemia, diabetes impairs bone regeneration and healing, resulting in a higher implant failure rate [6,7]. Therefore, effective interventions are needed to explore the underlying mechanisms of osseointegration in diabetes.

Alogliptin, a dipeptidyl peptidase-4 (DPP-4) inhibitor, has potent hypoglycemic effects, high bioavailability, and better specificity in vivo [8]. Alogliptin has proven to be the best medicinal cure in terms of bone metabolism [9], with many anti-diabetes medicines increasing fracture risk while alogliptin helps lower it to the greatest extent. Recent studies have found that glucagon-like peptide 1 (GLP-1) can stimulate the proliferation of osteoblasts and promote the expression of genes related to bone formation, but it is quickly degraded and inactivated by DPP-4 in vivo [10]. Different cell types, including osteoblast, BMSCs and osteoblast-like cells (MC3T3-E1) express GLP-1R, which triggers *cAMP/PKA/Wnt/β-catenin*, *PI3K/Akt* and *MAPK* pathways to upregulate *RUNX2* and osteogenic markers, driving mesenchymal cell differentiation into mineralized osteoblasts. Meanwhile, *GLP-1R* signaling elevates *OPG/RANKL* ratio and stimulates calcitonin secretion to suppress osteoclastogenesis and bone resorption, achieving net bone anabolism. However, alogliptin inhibits the activity of DPP-4 and prolongs the duration of GLP-1 treatment to fully exert the osteoprotective effect [11]. Alogliptin promoted MC3T3-E1 to differentiate and mineralize by upregulating osteogenic genes (including *RUNX2*, *OSX*, and *OCN*) and increasing ALP activity [12,13,14,15]. Interestingly, alogliptin is one of the hypoglycemic agents in clinical use, but whether it can promote osseointegration in diabetes is unknown. Our design of this experiment is to investigate the therapeutic effect and the mechanism of alogliptin in the osseointegration of diabetes, including clinical experiments in vitro and in vivo.

Though systemic alogliptin administration has been proven to enhance implant osseointegration in diabetic subjects, its clinical application still confronts the following obstacles. Specifically, with approval from endocrinologists, switching patients’ routine hypoglycemic medication to alogliptin during the bone healing phase can accelerate peri-implant bone regeneration and lower the risk of implant failure to a certain extent. Nevertheless, arbitrary switching of systemic hypoglycemic drugs is poorly tolerated by patients, resulting in low treatment compliance, elevated clinical risks, and a higher incidence of secondary complications such as hypoglycemia and fluctuating blood glucose levels. Against this backdrop, an increasing number of researchers have turned to biomaterial-based local drug delivery systems to circumvent the drawbacks associated with systemic medication adjustment. If locally delivered, alogliptin retains its capacity to boost diabetic osseointegration, a strategy that not only resolves the clinical challenge of compromised bone regeneration around implants in diabetic patients but also eliminates the systemic risks incurred by switching oral hypoglycemic agents. Such a therapeutic approach holds great translational significance for the clinical application of alogliptin to facilitate implant osseointegration among diabetic populations. GelMA was chosen as the drug carrier for its favorable biocompatibility, injectability and photocrosslinking capacity. Its porous structure enables sustained alogliptin release at the bone implant site, and its biodegradation rate matches the period of bone remodeling, avoiding the drawbacks of systemic medication.

In this study, we aim to determine whether alogliptin positively influences the oral osseointegration of people with diabetes and to establish an approach by which to improve stagnant immunomodulation, inhibited angiogenesis, and diminished osteogenesis in the diabetic bone microenvironment and achieve favorable bone regeneration.

## 2. Results

### 2.1. People with Diabetes Under DPP4i Medication Show Less Marginal Bone Resorption

#### 2.1.1. Included Case Information

A total of 13,179 cases from patients in the Department of Oral Implants at the Third Affiliated Hospital of Air Force Medical University from 2012 to 2022 were collected for this retrospective study. A total of 206 patients with 387 implants met the criteria. To minimize the influence of the implant system on the results of the experiments, Nobel Biocare (Gothenburg, Sweden), Straumann (Basel, Switzerland), and Takumi (Grenchen, Switzerland), which are commonly used in clinical practice, were selected. After excluding implants from other systems, a total of 156 patients with 286 implants met the inclusion criteria. To minimize errors caused by inconsistent implant positions, only patients with mandibular first molar teeth were collected, and a total of 138 patients met the study requirements.

The basic information about the patients is shown in Table 1. Among them, 45 patients were taking metformin, 42 patients were injecting insulin, 33 patients were injecting GLP-1 analogue, and 18 patients were taking DPP4 inhibitor. The possible factors affecting marginal bone resorption were first analyzed using one-way ANOVA, including patient age, HbA1c%, gender, implant platform level, implant brand and implant length. It was found that none of the above factors differed significantly between groups (*p* > 0.05) (Table 1). It was concluded that the above factors did not influence the main effects analysis.

#### 2.1.2. Comparison of Marginal Bone Loss

The results of marginal bone loss were different in each group (Table S1). The proximal and distal marginal bone loss 6 months after implant placement with DPP4 inhibitors was significantly lower than that in metformin and insulin. There was no statistically significant difference between the two medications, although the proximal and distal marginal bone loss values were less than those in the GLP-1 medication. In conclusion, Patients receiving DPP-4 inhibitors exhibited lower proximal and distal marginal bone loss, indicating a tendency toward improved peri-implant bone preservation compared with other hypoglycemic medications.

### 2.2. Systemic Application of Alogliptin Attenuates the Adverse Effects of Diabetes on Rats Implant Osseointegration

#### 2.2.1. Establishment of T2DM Rat Implant Model After Different Medications

**Figure 1:**
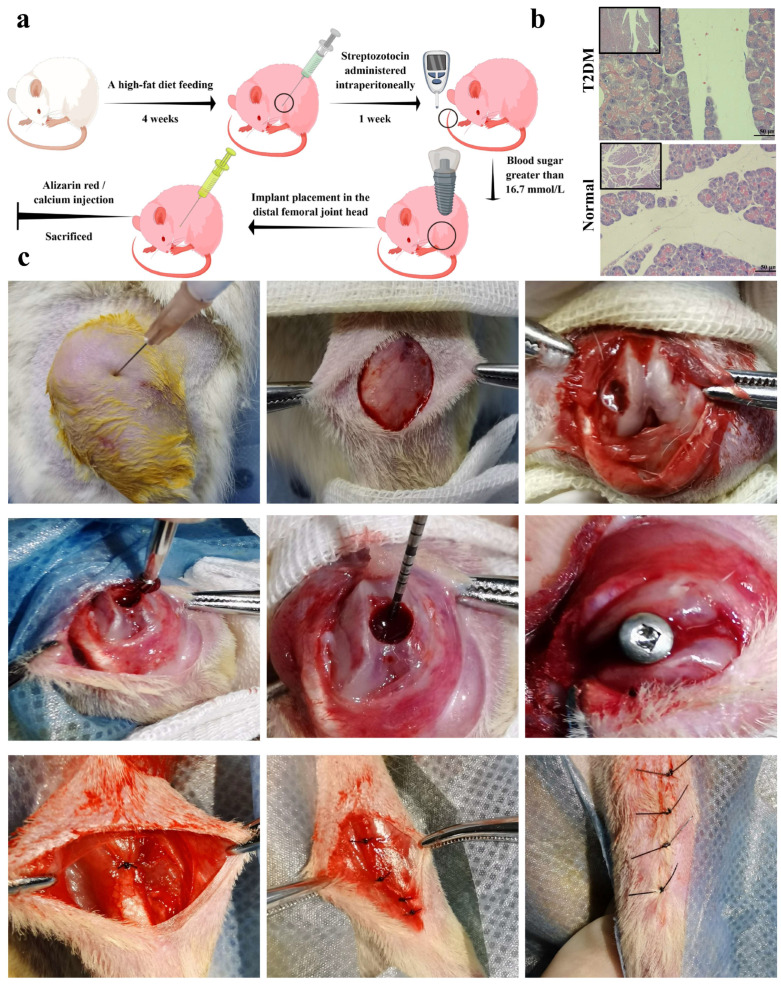
Establishment of T2DM rat model and implementation of implantation operation. (**a**) Schedule of study in vivo. (**b**). HE staining of islet tissue sections of normal rats and T2DM rats. (**c**) operation procedure of implant: The bilateral femoral skin and soft tissue were routinely disinfected and shaved, and a longitudinal incision was made at the posterior lateral femur, fully exposing the femoral intercondylar fossa. A standardized implant bed was carefully prepared at the predetermined position of the femoral intercondylar fossa. The prepared implant was precisely implanted into the femoral implant bed in place. Subcutaneous tissue and skin were sutured layer by layer.

To detect the effects of alogliptin on the osseointegration of diabetes rats, we designed the diabetes model by STZ injection after high-fat feeding for 4 weeks (Figure 1a). HE staining of the islet from diabetes rats represents a disordered state (Figure 1b). The surgical procedure is shown in Figure 1c.

#### 2.2.2. Effects of Systematic Application of Alogliptin on Implant Osseointegration in Diabetes

**Figure 2:**
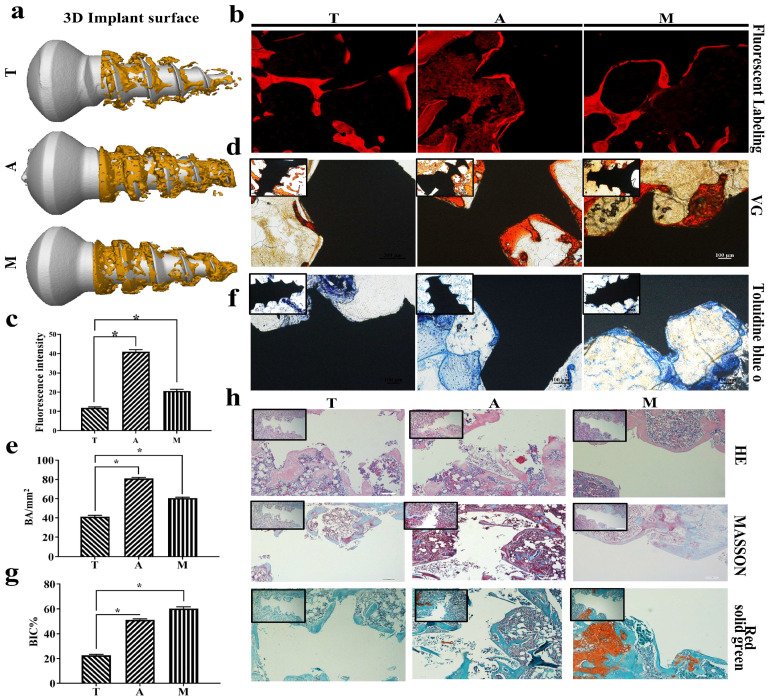
Alogliptin promotes greater bone regeneration than metformin in type II diabetic rats. (**a**) Bone healing in the different groups was determined by micro-CT at 4 weeks after implantation. (**b**) Images of fluorescence labelling observed under an immunofluorescence microscope. (**c**) Osteogenesis volumes were measured by fluorescent labelling. (**d**) Images of Van Gieson (VG) staining under the microscope. (**e**) Differences in the bone area (BA; peri-implant bone tissue ratio) were quantified by VG staining. (**f**) Images of Toluidine Blue (TB) staining under the microscope. (**g**) TB staining quantified differences in the BIC (bone–implant longitudinal contact surface length/total longitudinal implant length). (**h**) HE, modified Masson staining, Safranin O/Fast Green decalcified alogliptin and metformin systemic samples were used. T: T2DM control group; A: Alogliptin group; M: Metformin group (*n* = 7). * *p* < 0.05. Scale bar 1 cm:100 µm.

Results of micro-CT showed that alogliptin improved the osseointegration when compared with the control, while metformin enhanced the osseointegration (Figure 2a). The fluorescence experiment indicated that alogliptin represented a more intense fluorescence than the control, while metformin represented a similar trend (Figure 2b,c; comparison between T2DM control group (T) and alogliptin intervention group (A): * *p* = 0.024; comparison between T and metformin intervention group (M): * *p* = 0.031). VG and TB staining results showed that collagen fibers in the alogliptin group were clearer than control and metformin (Figure 2d,e, T vs. A: * *p* = 0.018; T vs. M: * *p* = 0.029). In contrast, muscle fibers in the alogliptin group were fewer than those in the control and metformin groups (Figure 2f,g, (Figure 2d,e, T vs. A: * *p* = 0.016; T vs. M: * *p* = 0.009)). HE, modified Masson and Safranin O/Fast Green staining demonstrated that alogliptin was more advantageous in promoting implant osseointegration in T2DM rats.

#### 2.2.3. Exploration of Systematic Application of Alogliptin on Potential Effective Pathways

**Figure 3:**
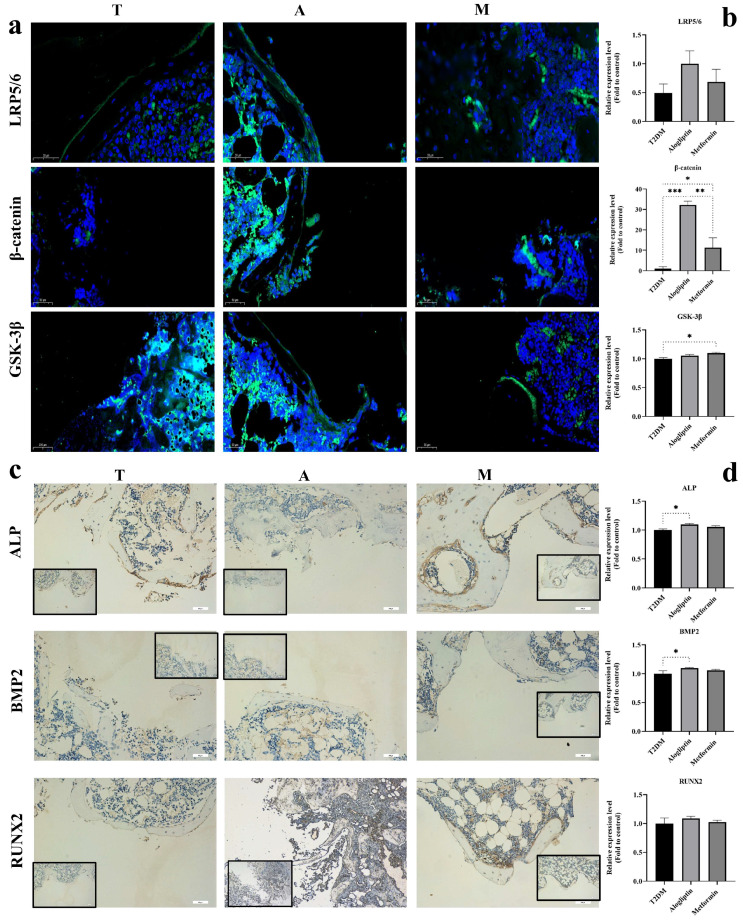
Greater osteo-inductivity of alogliptin is derived from activation of GLP1R/GSK-3β/β-catenin. (**a**) Immunofluorescent images of labelled GLP1R, β-catenin and GSK-3β around implants in the T2DM, alogliptin and metformin groups 4 weeks after the implant implantation in T2DM rats. (**b**) Statistical analysis of immunofluorescence. (**c**) Immunohistochemistry images labelled RUNX2, BMP2 and ALP around implants in the T2DM, alogliptin and metformin groups 4 weeks after the implant implantation in T2DM rats. (**d**) Statistical analysis of immunohistochemistry. T: T2DM control group; A: Alogliptin group; M: Metformin group (*n* = 7). * *p* < 0.05, ** *p* < 0.01, *** *p* < 0.001. Scale bars = 200 μm. The faint scale bar in the raw micrograph was retained to preserve the original imaging field of view; unified size calibration is defined in the caption.

The immunofluorescence results showed that alogliptin and metformin enhanced the expression of GLP1R (LRP5/6), β-catenin, and Gsk-3β (Figure 3a,b: β-catenin: T vs. A: *** *p* = 0.000; T vs. M: ** *p* = 0.008; A vs. M: * *p* = 0.023; *Gsk-3β*: T vs. A: A vs. M: * *p* = 0.034). Immunohistochemistry results showed that alogliptin and metformin enhanced the expression of *ALP*, *BMP2*, and *Runx2* (Figure 3c,d; *ALP*: T vs. A: * *p* = 0.028; *BMP2*: T vs. A: * *p* = 0.026), which means they each were able to improve the osteogenesis of BMSCs.

### 2.3. Local Alogliptin Delivery System Enhances Osseointegration by Improving the Diabetic Bone Microenvironment

#### 2.3.1. Establishment of the Alogliptin Delivery System

**Figure 4:**
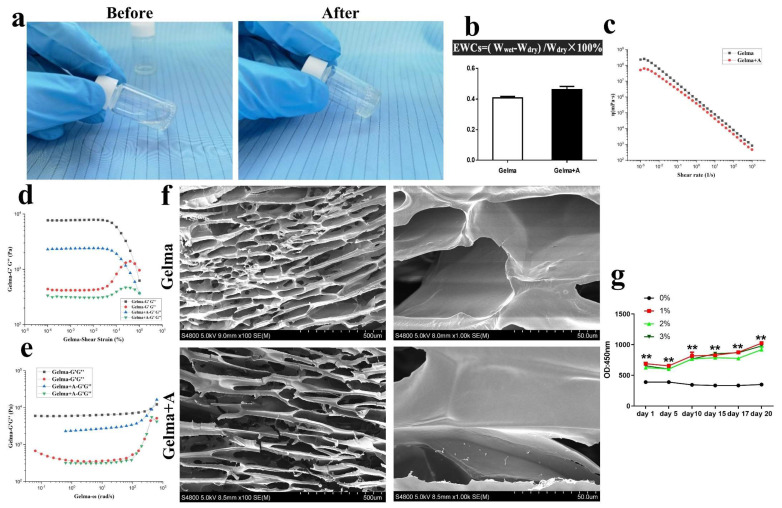
Characterization and identification of local alogliptin/BMSC delivery hydrogel system. (**a**) Scheme of hydrogel before and after coagulation. (**b**) The experiment of the equilibrium water volume, with the formula above. (**c**) Viscosity curve of different hydrogel groups. (**d**) Amplitude-scanned shear strain curves of different hydrogel groups. (**e**) Frequency-scanned shear strain curves of different hydrogel groups. (**f**) SEM images of the hydrogel with or without alogliptin. (**g**) Drug release of different concentrations of alogliptin through ELISA. GelMA: Blank delivery system; GelMA + A: Alogliptin delivery system. (*n* = 3). ** *p* < 0.01.

Combining the results of in vivo and in vitro experiments, we conclude that alogliptin is more suitable for the promotion of osseointegration in T2DM rats. To sustain the positive effects of alogliptin, we prepared gelatin methacryloyl (GelMA) hydrogel to deliver alogliptin (GelMA + A). Schemes of the hydrogel before and after solidification are shown in Figure 4a. Results of the equilibrium water volume experiment showed that the GelMA and GelMA + A maintained the same equilibrium water volume (Figure 4b). The viscosity test showed that the viscosity of the hydrogel decreased with an increasing shear rate, indicating a shear-induced disruption of the hydrogel network and demonstrating that the hydrogel was effectively shear-thinning and injectable (Figure 4c). The amplitude test in the rheological test demonstrated that the energy storage modulus (G′) and loss modulus (G″) of the hydrogel tend to equalize at close to 10% shear strain. At this point, the hydrogel transformed from a colloid to a fluid (Figure 4d). The frequency scanning results showed that G′ and G″ remained constant over a certain range. Then, both hydrogels tended to increase after reaching a certain frequency, reflecting the time-dependent viscoelastic characteristics of hydrogels (Figure 4e). SEM images illustrated GelMA + A assembled more drug granules and porous structure (Figure 4f). Results of ELISA showed that 1% alogliptin was the optimum concentration for the delivery system (Figure 4g; day1: 0% vs. 1%: ** *p* = 0.0072; 0% vs. 2%: ** *p* = 0.0085; 0% vs. 3%: ** *p* = 0.0061; day5: 0% vs. 1%: ** *p* = 0.0078; 0% vs. 2%: ** *p* = 0.0091; 0% vs. 3%: ** *p* = 0.0066; day10: 0% vs. 1%: ** *p* = 0.0046; 0% vs. 2%: **p = 0.0062; 0% vs. 3%: *p* = ** 0.0041; day17: 0% vs. 1%: **p = 0.0039; 0% vs. 2%: ** *p* = 0.0057; 0% vs. 3%: ** *p* = 0.0034; day20: 0% vs. 1%: ** *p* = 0.0028; 0% vs. 2%: ** *p* = 0.0043; 0% vs. 3%: ** *p* = 0.0022).

#### 2.3.2. Reversion of Alogliptin Delivery System on Poor Diabetic Implant Osseointegration

**Figure 5:**
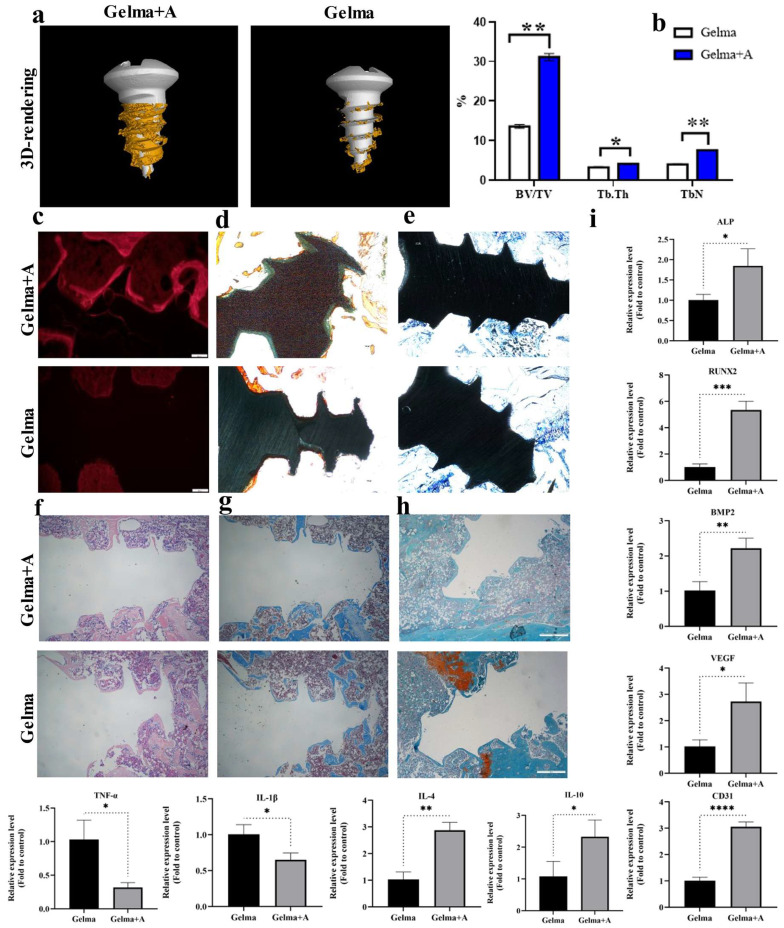
Local injection of alogliptin delivery system enhances diabetic rat osteogenesis through osteogenic–angiogenic immunomodulation. (**a**) Bone healing in the different groups was determined by micro-CT at 4 weeks after implantation. (**b**) The index of osseointegration of different groups. BV/TV: Percentage of bone volume; Tb. Th: Trabecular thickness; Tb. N: Trabecular number. (BV/TV: * *p* = 0.0032; Tb.Th: * *p* = 0.027; ** Tb.N: *p* = 0.0045). (**c**) Images of the double fluorescence labelling observed under an immunofluorescence microscope. (**d**) Images of Van Gieson (VG) staining under the microscope. (**e**) Images of Toluidine Blue (TB) staining under the microscope. (**f**) Images of HE staining under the microscope. (**g**) Images of Masson staining under the microscope. (**h**) Images of Safranin O/Fast Green under the microscope. (**i**) Analysis of qPCR of relative ALP (* *p* = 0.031), *RUNX2* (*** *p* = 0.0007), *BMP2* (** *p* = 0.0042), *VEGF* (* *p* = 0.025), *CD31* (**** *p* = 0.0000), TNF-α (* *p* = 0.0028), *IL-1β* (* *p* = 0.0034), *IL-4* (** *p* = 0.0051) and *IL-10* (* *p* = 0.022) levels. GelMA: Blank delivery system; GelMA + A: Alogliptin delivery system. (*n* = 7). * *p* < 0.05, ** *p* < 0.01, *** *p* < 0.001, **** *p* < 0.0001. Scale bars = 200 μm. The faint scale bar in the raw micrograph was retained to preserve the original imaging field of view; unified size calibration is defined in the caption.

**Figure 6:**
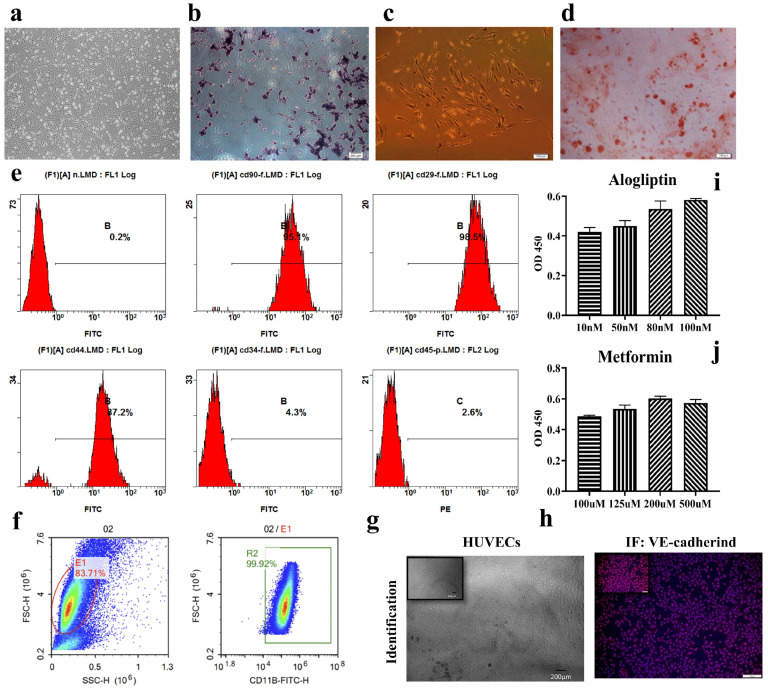
Cell identification and drug concentration. (**a**) Image of bone marrow mesenchymal stem cells (BMSCs) under microscope, scale bar = 200 μm. (**b**) Osteogenesis detected by ALP. (**c**) Adipogenesis detected by Oil Red. (**d**) Osteogenesis detected by Alizarin Red. (**e**) Identification of BMSCs through flow cytometry. (**f**) Identification of BMMs through flow cytometry. (**g**) Images of HUVECs under a microscope, scale bar = 200 μm. (**h**) Immunofluorescent staining of HUVECs stained with VE-cadherin, scale bar = 200 μm. (**i**) Drug concentration analysis of BMSC on alogliptin. (**j**) Drug concentration analysis of BMSC on metformin. (*n* = 3). Scale bars = 200 μm. The faint scale bar in the raw micrograph was retained to preserve the original imaging field of view; unified size calibration is defined in the caption.

GelMA hydrogel represents an outstanding biomaterial-based drug delivery system for local peri-implant treatment. Micro-CT showed that the GelMA + A group could sustainably improve diabetic implant osseointegration compared with GelMA group (Figure 5a). The indices, including BV/TV (*p* = 0.003), Tb.TH (*p* = 0.048) and TbN (*p* = 0.000), were consistent with the trend of Figure 5a,b. The fluorescence experiment indicated that GelMA + A represented a more intense fluorescence than control (Figure 5c). Results of VG and Toluidine Blue showed that collagen fibers in the alogliptin group were much clearer than control (Figure 5d,e). In decalcified sections, HE (Figure 5f) showed that inflammatory cells were more sparsely distributed and fewer in number after GelMA + A intervention. Modified Masson’s staining (Figure 6g) showed that, though less red-stained mature bone tissue was formed after GelMA + A intervention, Safranin O/Fast Green pre-osteogenic collagen fibers were more evenly distributed over a wider area. Safranin O/Fast Green (Figure 5h) showed that a greater proportion of blue-stained bone tissue was formed after GelMA + A intervention, especially in the implant root region, which is important for solid osseointegration. This suggested that GelMA + A promoted new bone production, whereas the mature bone in the GelMA group may have been pre-existing aged bone. Overall, the GelMA + A group was more advantageous in promoting osteointegration of implants in T2DM rats. After extracting RNA from peri-implant bone tissue, its qPCR results showed that the GelMA + A group promoted the transcription of more osteogenesis-related genes, more angiogenesis-related genes, and more anti-inflammation-related genes (Figure 6i).

### 2.4. Mechanism Exploration of Alogliptin Induced Osteogenesis, Angiogenesis and Immunomodulation

#### 2.4.1. Cell Identification and Exploration of Appropriate Concentration of Hypoglycemic Medication on BMSCs

BMSCs were seen as cords of scattered cells under the microscope (Figure 6a). The potential for multinomial differentiation was detected by ALP (Figure 6b), Oil Red (Figure 6c) and Alizarin Red (Figure 6d). BMSCs expressed specific surface biomarkers, which were 95.1% CD90, 98.5% CD29 and 87.2% CD44, while stem cell biomarkers like CD34 (4.3%) and CD45 (2.6%) were rarely seen on BMSCs (Figure 6e). With regard to live BMMs, 99% of them expressed CD11b, confirming the purity of BMMs (Figure 6f). HUVECs showed their starlike shape under the microscope (Figure 6g) and the results of immunofluorescence showed their over-expression of VE-cadherin (Figure 6h). The optimum drug concentration of alogliptin was 100 nM (Figure 6i) and was 200 μM for metformin (Figure 6j).

#### 2.4.2. Effects of Alogliptin on the Behaviors of BMSCs, BMMs and HUVECs

**Figure 7:**
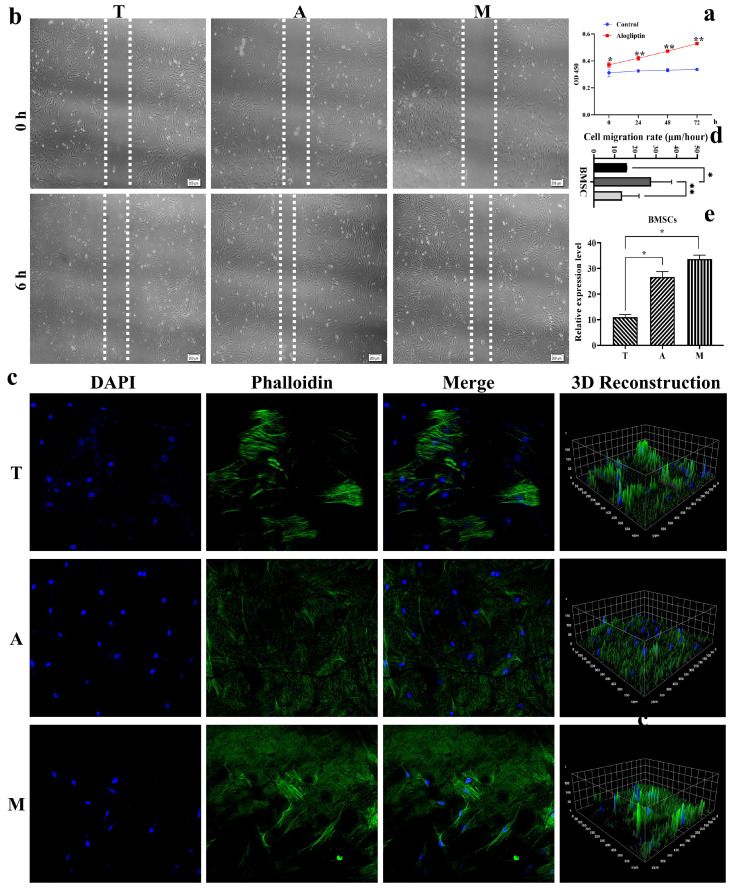
Alogliptin offers greater potential than metformin to establish the implant–bone connection by promoting BMSC viability, migration and adhesion. (**a**) Proliferation of BMSCs after 0 h, 24 h, 48 h, and 72 h was determined by CCK-8 assays. (**b**) Differences in the migration ability of BMSCs. (**c**) Differences in the adhesion ability of BMSCs. (**d**) Statistical analysis of the differences in the migratory capacity of the above cell type. (**e**) Statistical analysis of the differences in the adhesion capacity of the above cell type. Control: PBS intervention; Alogliptin: Alogliptin intervention. T: T2DM control group; A: Alogliptin group; M: Metformin group. (*n* = 3). * *p* < 0.05, ** *p* < 0.01. Scale bars = 200 μm. The faint scale bar in the raw micrograph was retained to preserve the original imaging field of view; unified size calibration is defined in the caption.

**Figure 8:**
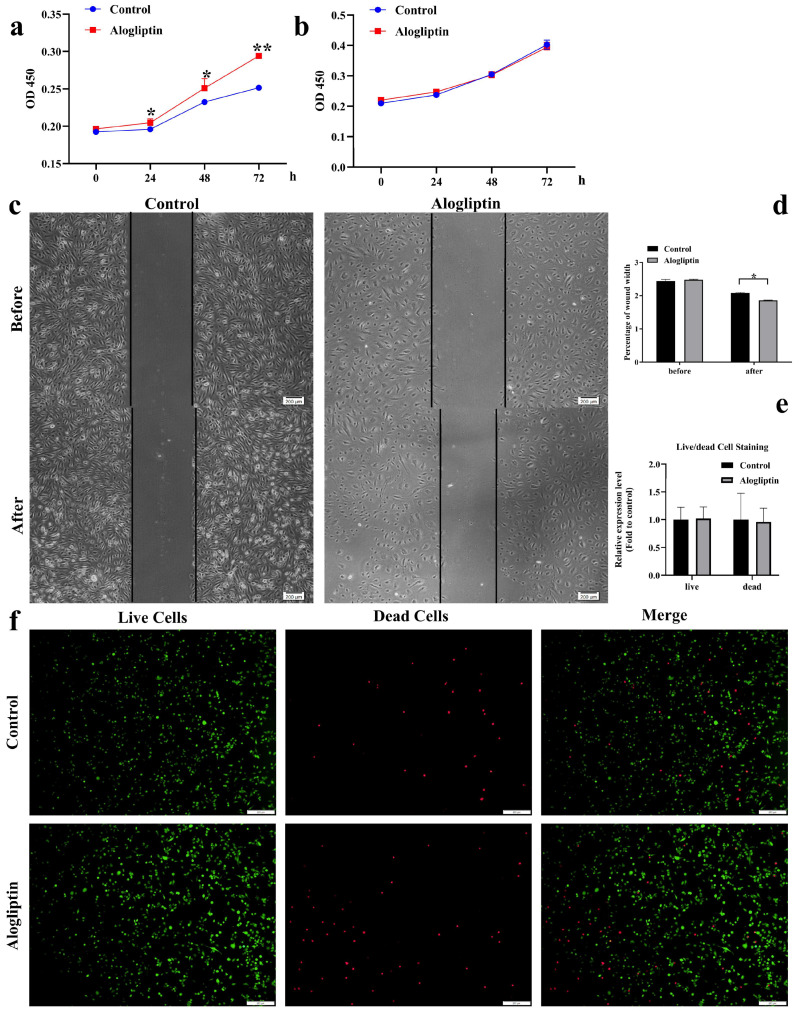
Biosafety of alogliptin on HUVECs and BMMs. (**a**) Proliferation of HUVECs after 0 h, 24 h, 48 h, and 72 h was determined by CCK-8 assays. (**b**) Proliferation of BMMs after 0 h, 24 h, 48 h, and 72 h was determined by CCK-8 assays. (**c**) Scratch wound healing of HUVECs cocultured with different groups after 6 h, scale bar = 200 μm. (**d**) Quantitative analysis of scratch wound healing. (**e**) Statistical analysis of live/dead cell staining. (**f**) Images of live/dead cell staining. Control: PBS intervention; Alogliptin: Alogliptin intervention. (*n* = 3). * *p* < 0.05, ** *p* < 0.01. Scale bars = 200 μm. The faint scale bar in the raw micrograph was retained to preserve the original imaging field of view; unified size calibration is defined in the caption.

CCK-8 assay showed a faster proliferation in BMSCs after alogliptin and metformin intervention; (Figure 7a, control (T) vs. alogliptin (A): 0 h: * *p* = 0.033; 24 h: ** *p* = 0.0071; 48 h: ** *p* = 0.0056; 72 h: ** *p* = 0.0039; control (T) vs. metformin (M): 0 h: * *p* = 0.217; 24 h: ** *p* = 0.003; 48 h: ** *p* = 0.0004; 72 h: ** *p* = 0.0016). Results of wound healing showed that alogliptin and metformin enhanced the migration of BMSCs after 6 h (Figure 7b,d; T vs. A: * *p* = 0.029; T vs. M: * *p* = 0.032; M vs. A: *p* = 0.0068). Immunofluorescence results showed that alogliptin and metformin enhanced the adhesion of BMSCs after 6 h (Figure 7c,e; T vs. A: * *p* = 0.024; T vs. M: * *p* = 0.017; M vs. A: *p* = 0.0038). CCK-8 assay also showed a faster proliferation in HUVECs and BMMs after alogliptin intervention (Figure 8a,b; control (T) vs. alogliptin (A): 0 h: *p* = 0.196; 24 h: * *p* = 0.034; 48 h: * *p* = 0.0027; 72 h: ** *p* = 0.0041). Then, the scratch experiment also showed faster tissue healing after alogliptin intervention (Figure 8c,d). Live and dead cell experiments showed that after 7 days of alogliptin intervention, alogliptin had little toxicity to BMMs (Figure 8e,f).

#### 2.4.3. Effects of Alogliptin on Osteogenic–Angiogenic–Immunomodulatory Functions Triggered by BMSCs

**Figure 9:**
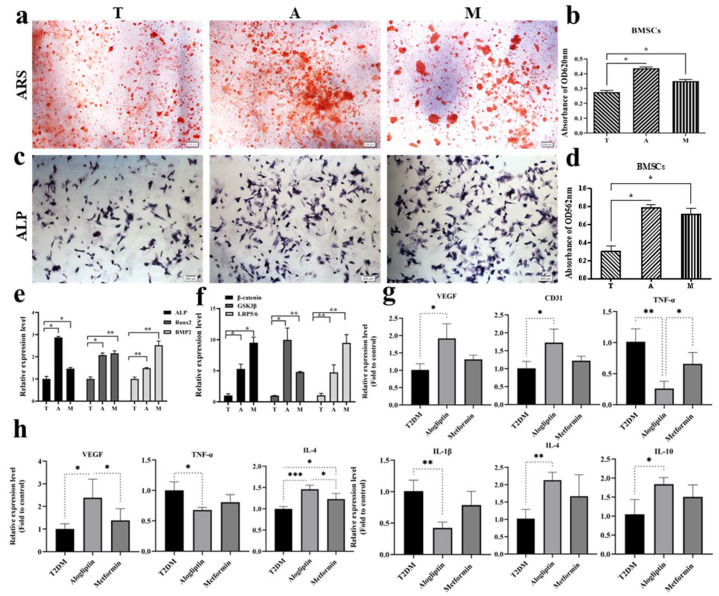
Effect of alogliptin on osteogenesis and osteogenic–vascular immune-coupling-related gene in BMSCs. (**a**) Alizarin Red assay was used to identify the difference in osteogenic differentiation ability of the above cell type. (**b**) Statistical analysis of the osteogenic differentiation ability of the above cell type. (**c**) ALP assay was used to identify the difference in osteogenic differentiation ability. (**d**) Statistical analysis of the osteogenic differentiation ability of the above cell type. (**e**) Analysis of qPCR of differences in expression of osteogenic-related genes. (**f**) Analysis of qPCR of differential expression of *Wnt/β-catenin* pathway. (**g**) Analysis of qPCR of angiogenic-related or inflammatory-related genes. (**h**). Analysis of ELISA of angiogenic-related or inflammatory-related proteins. T: T2DM control group; A: Alogliptin group; M: Metformin group. (*n* = 3). * *p* < 0.05, ** *p* < 0.01, *** *p* < 0.001. Scale bars = 200 μm. The faint scale bar in the raw micrograph was retained to preserve the original imaging field of view; unified size calibration is defined in the caption.

ALP and Alizarin Red results showed that alogliptin and metformin expressed more ALP and calcium nodules of BMSCs (Figure 9a–d; ARS:T vs. A: * *p* = 0.025; T vs. M: * *p* = 0.031; ALP: T vs. A: * *p* = 0.025; T vs. M). Results of qPCR showed that alogliptin and metformin improved the expression of *ALP*, *Runx2* and *BMP2* (Figure 9e; ALP:T vs. A: * *p* = 0.026; T vs. M: * *p* = 0.036; Runx2: T vs. A: * *p* = 0.084; T vs. M: * *p* = 0.016: BMP2: T vs. A: * *p* = 0.024; T vs. M: ** *p* = 0.0087). Results of qPCR showed that alogliptin and metformin enhanced the expression of *GSK3β*, *LRP5/6* and *β-catenin* (Figure 9f; *GSK3β*: T vs. A: ** *p* = 0.084; T vs. M: ** *p* = 0.0046; *LRP5/6*: T vs. A: ** *p* = 0.0072; T vs. M: ** *p* = 0.0016: *β-catenin*: T vs. A: * *p* = 0.034; T vs. M: * *p* = 0.018), which indicated that alogliptin and metformin improved osteogenesis by the *Wnt/β-catenin* pathway. Analysis of qPCR and ELISA of angiogenic-related or inflammatory-related genes and cytokines of co-cultured HUVECs and M1-BMMS demonstrated that alogliptin can promote angiogenesis immunomodulation by the paracrine mechanism of BMSCs (Figure 9g,h; *VEGF*: T vs. A: ** *p* = 0.084; T vs. M: ** *p* = 0.0046; *CD31*: T vs. A: ** *p* = 0.0072; T vs. M: ** *p* = 0.0016: *TNF-α*: T vs. A: * *p* = 0.034; T vs. M: * *p* = 0.018; IL-1β: T vs. A: ** *p* = 0.0076; T vs. M: *p* = 0.121; IL-4: T vs. A: *** *p* = 0.0072; T vs. M: * *p* = 0.022; IL10:T vs. A: ** *p* = 0.0022; T vs. M: *p* = 0.093).

#### 2.4.4. Angiogenesis and Immunomodulation of Alogliptin on HUVECs and M1-BMMs

**Figure 10:**
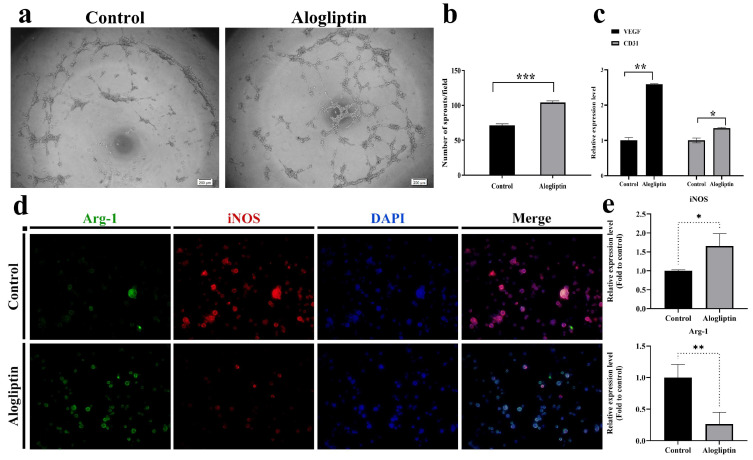
Alogliptin promotes angiogenesis in HUVECs and anti-inflammation in M1-BMMS. (**a**) Images of tube formation after different interventions. (**b**) Quantitative analysis of tube formation of HUVECs; scale bar = 40 μm. (**c**) Analysis of qPCR of relative *VEGF* and *CD31* mRNA levels in HUVECs. (**d**) Immunofluorescent level of *iNOS* and *Arg-1* expression. (**e**) Analysis of the relative immunofluorescent value of *iNOS* and *Arg-1*. Control: PBS intervention; Alogliptin: Alogliptin intervention. (*n* = 3). * *p* < 0.05, ** *p* < 0.01, *** *p* < 0.001. Scale bars = 200 μm. The faint scale bar in the raw micrograph was retained to preserve the original imaging field of view; unified size calibration is defined in the caption.

Results of the tube formation assay showed more angiogenesis after alogliptin intervention (Figure 10a,b; number of sprouts/field: Control vs. alogliptin: *** *p* = 0.0008). qPCR showed that alogliptin over-expressed the angiogenesis genes, including *VEGF* and CD31(Figure 10c; control vs. alogliptin: *VEGF*: ** *p* = 0.0041; *CD31*: * *p* = 0.0025). Meanwhile, the effect of alogliptin also involved the immunomodulation of M1-BMMs. The results of immunofluorescence experiments demonstrated that alogliptin promoted the anti-inflammatory modulation of M1-BMMs and inhibited their pro-inflammatory transformation (Figure 10d,e; control vs. alogliptin: *iNOS*: * *p* = 0.029; Arg-1: ** *p* = 0.0063).

## 3. Discussion

DM was first considered an absolute contraindication to implant restoration because its unstable blood glucose level affected bone metabolism, leading to the accumulation of ROS in the body and exacerbating the inflammatory response [16]. With the progress of surgical techniques and implant surface treatment, ever more clinicians are beginning to believe that the success rate of the implant recovery of people with diabetes with stable blood glucose is consistent with that of those without diabetes [17]. Therefore, finding the best way to control blood glucose is of great practical significance in solving the clinical problem of low diabetic osseointegration success rate [18]. If such drugs have a more positive bone metabolism effect, it will further improve the patient’s therapeutic efficacy and achieve the purpose of dual treatment of DM with low efficiency of osseointegration. The results of clinical case studies suggest that the four most common types of clinical glucose-lowering drugs include metformin, insulin, GLP1, and DPP4 inhibitors, among which the long-term marginal bone resorption values of patients taking DPP4 inhibitors are significantly lower than those of other drug groups, suggesting that they have an optimal bone metabolism effect.

Our clinical retrospective cohort analysis revealed that T2DM patients treated with DPP4 inhibitors exhibited the lowest marginal bone resorption after dental implantation when compared with patients receiving metformin, insulin, or GLP-1 analog therapy. To further interpret the superior peri-implant bone preservation ability of DPP4 inhibitors, we propose mechanistic hypotheses based on their distinct pharmacological characteristics, particularly explaining why DPP4 inhibition yields more stable osseointegration outcomes than GLP-1 analog intervention. Although both strategies activate GLP-1R signaling and contribute to bone anabolism, their activation patterns differ fundamentally. GLP-1 analogs deliver exogenous, intermittent pharmacological stimulation, which induces transient pathway activation and fluctuating osteogenic activity; such short-term stimulation is insufficient to sustain continuous bone regeneration during the long osseointegration remodeling cycle under diabetic conditions. In contrast, DPP4 inhibitors exert persistent endogenous regulatory effects by blocking the DPP4-mediated degradation of intrinsic GLP-1 and GIP, thereby prolonging the half-life of endogenous incretins and maintaining stable, long-term GLP-1R signaling activation. This sustained GLP-1R/GSK3β/β-catenin cascade activation effectively enhances BMSC osteogenic differentiation, facilitates peri-implant angiogenesis, and restrains M1-type proinflammatory macrophage polarization, collectively remodeling the compromised diabetic bone microenvironment. Moreover, metformin improves diabetic bone conditions mainly through systemic hypoglycemic regulation without specific targeting of bone metabolic signaling, while alogliptin possesses dual advantages of metabolic regulation and direct bone protective bioactivity. By simultaneously coordinating osteogenesis, angiogenesis, and immune homeostasis, alogliptin treatment achieves more durable marginal bone maintenance and superior implant osseointegration in diabetic patients.

Alogliptin is a DPP4 inhibitor. It achieves the effect of glycemic reduction by inhibiting the inactivation of DPP4 and gastric inhibitory polypeptide (GIP) and increasing the expression of endogenous GLP-1, promoting insulin secretion from pancreatic β-cells and inhibiting glucagon secretion [19]. Compared with the initial subjects, who treated their diabetes with metformin alone, the serum levels of osteocalcin (OC) and type I collagen amino-terminal prolongation peptide (PINP) were significantly increased after 16 weeks of treatment with metformin in combination with alogliptin, confirming its bone-enhancing clinical effect [20]. The possible mechanism of this effect is related to the elevation of serum Apelin-13 level by alogliptin. In addition to this, DPP4 inhibitors are safer in terms of bone metabolism [21]. For example, rosiglitazone increases bone loss and fracture risk, dapagliptin stimulates bone resorption, and sitagliptin indirectly raises fracture risk [9]. Not coincidentally, a recent study confirmed that alogliptin has the most positive effect on bone metabolism in DPP4-IV medicine, better than sitagliptin [22]. Combined with the results of the group’s preclinical study (DPP4 inhibitors are, among the more hypoglycemic drugs, the drugs with the best protective effect on marginal bone resorption in people with diabetes), these clinical observations imply that alogliptin may confer superior bone-protective effects among commonly used anti-diabetic drugs, which warrants further prospective verification. Its optimal promotion of diabetic implant osteointegration has also been verified by the above-mentioned experiments, which, on the other hand, confirms its optimal bone metabolism effect.

Osseointegration is the direct union of bone tissue and titanium, with no fibrous tissue growing in between [23]. Its biological process includes foreign body reaction after implantation, where the foreign body will activate the body’s immune system and gather a large number of phagocytes around the implant, with the phagocytes being absorbed or macrophages polarized to the M2 type. In addition, the biological process of osseointegration includes the activation of vascularization around the implant and the process whereby a large number of newly generated blood vessels provide a framework and nutrients for osteoclasts and BMSCs, promoting their osteogenesis so as to differentiate and gradually form mature bone tissues around the implant. Numerous studies have demonstrated that DM adversely affects these biological processes, including the tendency of macrophages to convert to the M1 type, the decline of angiogenic function, and the weakening of the osteogenic differentiation ability of BMSCs [24,25,26]. People with diabetes have significantly higher failure rates of implant restorations, more long-term marginal bone resorption and more severe peri-implantitis than non-diabetes [17,27]. The results of our single-center retrospective study also found that people with diabetes had significantly more marginal bone resorption than non-diabetics. The experimental results of our data analysis, which included glucose-lowering medication as the only variable, found that alogliptin was the best inhibitor of marginal bone resorption. This suggests that alogliptin is the most active bone metabolizing drug among the clinically used hypoglycemic drugs.

There are a variety of signaling pathways involved in the process of osteogenesis. Direct targeting of osteogenesis, such as *Wnt/β-catenin*, drives osteoblast differentiation and osteogenesis [28]. The main mechanism is to target the cell membrane receptor (*LRP5/6*) through Wnt protein (Frizzled), which inhibits *β-catenin* degradation in the cytoplasm, resulting in *β-catenin* entry into the nucleus and activating the expression of osteoblast-related genes to promote osteogenesis or BMSC osteogenesis. In addition, some signaling pathways indirectly promote osteogenesis by targeting osteoclast function. Classically, this is *osteoprotegerin (OPG)*, which is secreted by osteoclasts as a ‘decoy receptor’ and competitively binds to *RANKL*, thus blocking the interaction between RANKL and RANK to inhibit osteoclastogenesis [29]. Denosumab, which is commonly used in the clinic, plays a role in the treatment of osteoporosis by inhibiting *RANKL*. Currently, there is no evidence to confirm that alogliptin directly regulates the *Wnt/β-catenin* signaling pathway [30]. Our expression data demonstrated upregulated GLP1R, GSK-3β and β-catenin following alogliptin intervention, implying potential involvement of the *GLP1R/GSK-3β/β-catenin* cascade in mediating its osteogenic effects. However, without GLP1R inhibition or β-catenin knockdown rescue experiments, we cannot confirm that alogliptin relies exclusively on this pathway to improve diabetic osseointegration. At present, no published evidence has validated direct regulatory interactions between alogliptin and the Wnt/β-catenin axis. Injectable hydrogels have become the focus of attention in bone regeneration due to their superior drug-carrying properties, biocompatibility, and high clinical conversion rate. In this regard, a hydrogel is capable of successively inducing chondrogenesis and endochondral osteogenesis for bone regeneration with osteogenic-vascularization coupling through the chelation of iron ions in the original flavor and the creation of a low-oxygen environment [31]. However, it remains doubtful whether osteogenesis within chondrogenesis is more effective than direct osteogenesis. Moreover, whether the hypoxic environment favors bone remodeling after initial osteogenesis and whether it induces changes in the immune microenvironment remains unknown. There is also a hydrogel that mimics the extracellular matrix and promotes blood vessel formation and bone formation by enhancing the release of silicon and magnesium ions [32]. However, the targeting of osteogenesis and vascularization is still lacking. Therefore, benefiting from the modulatory advantages of alogliptin in osteogenesis, angiogenesis and immunomodulation, we designed a hydrogel that improved implant osseointegration in diabetic rats.

Several limitations of the present study should be acknowledged to objectively evaluate the translational value of the findings. First, the clinical investigation was performed as a single-center retrospective study, which inevitably introduces inherent selection bias and limits population representativeness. Notably, the present single-center retrospective clinical analysis cannot confirm causal effects between alogliptin treatment and attenuated marginal bone loss, and only reveals a statistical association. The small sample size of the DPP-4 inhibitor group represents a key limitation, and confounding clinical variables were further adjusted via multivariable regression in the revised dataset. For this reason, we avoid definitive claims that alogliptin is the superior agent for bone metabolism; long-term, large-scale prospective clinical studies are essential to validate the causal osteoprotective benefits of DPP-4 inhibitors in diabetic patients receiving dental implants. Second, although we detected altered expression of GLP1R/GSK-3β/β-catenin signaling molecules after alogliptin administration, this study did not conduct receptor blockade, gene knockdown or rescue experiments to functionally verify the necessity of this pathway. Therefore, we can only propose a correlative association rather than a definitive causal mechanism linking alogliptin to Wnt/β-catenin activation. Third, the injectable GelMA hydrogel drug delivery system was only validated in a rodent femoral implant model. Given the interspecies differences in bone structure, sugar metabolism level, and local immune microenvironment, further verification in higher-order animal models is needed to optimize its clinical transformability. Fourth, the current in vivo evaluation focused only on an 8-week observation period, which mainly reflects early and middle stages of osseointegration. Long-term hydrogel degradation behavior, sustained drug release stability, and late peri-implant bone remodeling still require prolonged experimental monitoring.

We have developed an alogliptin delivery system which demonstrated that alogliptin induces osteogenic differentiation of BMSCs and promotes angiogenic and anti-inflammatory effects independently or through BMSCs. However, the regulation of angiogenesis and anti-inflammation at different stages of osteogenic differentiation of BMSCs induced by alogliptin is still uncertain. We hope that more studies will look deeper to analyze the effects of alogliptin-induced angiogenesis and anti-inflammation in different stages of osteogenic differentiation of BMSCs by spatiotemporal transcriptomics or other technologies that can shed light on this issue.

## 4. Materials and Methods

### 4.1. Retrospective Cohort Study

#### 4.1.1. Study Design and Patient Selection

This retrospective study was conducted in the Department of Oral Implants at the Third Affiliated Hospital of Air Force Medical University from 2012 to 2022. The study followed the Strengthening the Reporting of Observational Studies in Epidemiology (STROBE) statement and was ethically approved by the Ethics Committee of the Third Affiliated Hospital of Air Force Military Medical University (2021133). Patients were excluded because they had changed their hypoglycemic medication before or during oral implant treatment; had severe bone metabolic diseases such as osteoporosis or long-term use of bisphosphonates; smoked cigarettes or chewed betel quid; had severe DM complications or other serious illnesses; had overdenture prostheses on the implant; and were unable to attend follow-up appointments on time or were inaccessible for contact. Included patients were required to meet the following criteria: confirmed diagnosis of T2DM with a stable disease process; preoperative HbA1c% <8.0% and no other complications; no change of hypoglycemic medication; no bone augmentation surgery was used in the treatment; the restoration was a fixed prosthetic modality; and no mucosal or periodontal diseases or occlusal disorders.

#### 4.1.2. Data Collection

The imaging data collected immediately after implant surgery and at 6 months postoperatively included cone-beam computed tomography (CBCT), apical films and digitized full-mouth surface tomograms. The marginal bone height of the implants at the two different stages was measured and recorded using Digimizer v 5.8.0 (Med Calc Inc., Ostend, Belgium) medical measurement software, with three measurements taken at each time and averaged. In order to reduce systematic errors, the contact point between the proximal and distal middle of the implant and the most coronal aspect of the alveolar ridge was firstly marked, and then the vertical distance from the apical apex of the implant to the contact point of the alveolar ridge was measured, and this distance was taken as the value of the marginal bone height of the implant. This was undertaken, instead of measuring the length of the implant’s shoulder plateau and the alveolar ridge directly, so as to avoid any measurement errors due to small distance. At the same time, the actual length of the implant was added as a scale to correct the measurement as well as the image deviation during the analysis. The formula for calculating the marginal bone height of the implant is as follows: Actual marginal bone height of the implant = Imaging implant marginal height × Implant length/Imaging implant length. Bone heights were calculated immediately after implant placement (baseline) and 6 months after implant placement to compare the effects of different medications on implant marginal bone resorption. All clinical indicators were assessed in a randomized grouping design. Fixed prostheses were applied for implant restoration in all patients at 6 months postoperatively.

### 4.2. Hydrogel

The Engineering For Life company (Suzhou, China) purchased commercial hydrogel and designed it according to instructions. A proper concentration of alogliptin was dissolved into GelMAEFL-GM-60 and used for the next experiments. Then, BMSCs were cross-linked with the hydrogel by solution immersion. The rheological characteristics of the hydrogel were evaluated using a rheometer. The viscosity of the hydrogel decreased with an increase in shear rate. GelMA hydrogel was prepared using LAP as the photoinitiator. Briefly, the hydrogel precursor solution consisted of 50 mg GelMA powder dissolved in 1 mL LAP-containing PBS solution (0.05 g LAP in 20 mL 1 × PBS). After full dissolution via heating and shaking under dark conditions, the solution was sterilized with a 0.22 μm filter for standby application. The alogliptin-loaded GelMA (Gel-A) hydrogel was prepared with identical matrix components as pure GelMA hydrogel. The Gel-A precursor system was composed of 50 mg GelMA and 7.5 mg alogliptin co-dissolved in 1 mL LAP PBS solution, with a fixed alogliptin loading dosage of 7.5 mg/mL in the final hydrogel. The mixed solution was fully dissolved in a dark environment and sterile-filtered through a 0.22 μm filter for subsequent local use.

#### 4.2.1. Equilibrium Water Volume Experiment

Amounts of 1 mL for both GelMA and GelMA + A (the GelMA combined with alogliptin and BMSCs) were UV-photocured and placed in a small dish. The weight was measured at this time. Then, they were put into a vacuum freeze-drying machine and the weight was measured again after 24 h, setting three sub-holes for each group.

#### 4.2.2. SEM

Amounts of 1 mL for both GelMA and GelMA + A were UV-photocured and placed in 6-well plates at −80 °C overnight. The next day, the 6-well plates were removed and placed in a vacuum freeze-drying machine for 48 h. Then, the hydrogel was cut into about 1 mm^3^, glued to a special tank for electron microscopy, and put into a sand blasting machine for gold spraying. The surface morphology, particle size distribution, spatial structure and other parameters of the hydrogel were then observed by scanning electron microscopy (Siemens, Munich, Germany).

#### 4.2.3. ELISA for Hydrogel

For the hydrogel, after light curing, 1 mL amounts of GelMA and GelMA + A were placed into 15 mL centrifuge tubes and added into each centrifuge tube before 10 mL 1 × PBS (pH = 7.4) solution was into an incubator at 37 °C and shaken. After 15, 17 and 20 days, the sustained-release system from the centrifuge tube was centrifuged at 6000 rpm for 5 min, and the supernatant was removed and stored in a −80 °C refrigerator. The centrifuge tube was filled with an equal amount of 1 × PBS. After all supernatant was collected, it was photocured with 1 mL GelMA and GelMA + A, then put into a 15 mL centrifuge tube, and added to each centrifuge tube. All supernatants are collected.

The prepared GelMA-based hydrogel exhibited a sustained local residence time of at least 8 weeks at the femoral implant site, matching the experimental observation cycle of this animal study.

### 4.3. In Vivo Study

#### 4.3.1. Induction of T2DM Rat Model

All procedures were approved by the Animal Ethics Committee of the Stomatology of the Fourth Military Medical University (Shaanxi, China) (Appl. No. kq-2022-011) and complied with the ARRIVE guidelines following the U.K. Animals (Scientific Procedures) Act, 1986 and associated guidelines, as well as EU Directive 2010/63/EU. Forty male Sprague–Dawley (SD) rats aged 12–13 weeks weighing 300 ± 30 g (supplied from the Chengdu Dossy Experimental Animal) were selected. The sample size was calculated by G*Power 3.1 software based on the core outcome indicators of bone osseointegration in T2DM rats. With α = 0.05 and power = 0.8, the theoretical minimum sample size was 6 rats per group. Considering the possible sample attrition caused by diabetes modeling, surgical operation and postoperative complications, we reserved individual animals with a 15% estimated loss rate, and finally set 7 rats in each group. After 1 week of adaptive feeding, T2DM rats were induced by a 4-week high-carbohydrate and high-fat diet (DOSSY, Chengdu, China), followed by intraperitoneal injection with streptozotocin (STZ, Sigma, St. Louis, MO, USA) at a dose of 35 mg/kg [33]. Rats with fasting glucose higher than 16.7 mmol/L stable for one week, were validated as T2DM and used for the next experiments.

#### 4.3.2. Surgery and Treatment

T2DM rats were randomly divided into three groups (T: Type II diabetes group, A: Alogliptin group, M: Metformin group) in the first stage and two groups (GelMA: Hydrogel delivery system, GelMA + A: Alogliptin hydrogel delivery system) in the second stage, and the number of rats in each group equals 7. SD rats meeting glycemic criteria were anesthetized with 1% pentobarbital sodium (45 mg/kg) for general anesthesia and Primacaine^®^ (Pierre Rolland, Bordeaux, France) for intraoral local analgesia. As previously described, implants were implanted into rats’ femur during the first animal experiment. An amount of 45 mg/kg of pentobarbital sodium was utilized for anesthesia and an implant bed was made on the femoral intercondylar fossa. The implants (3 × 1.5 mm, Shandong Hengtai Medical Device, Tai’an, China) were inserted and the subjects tightly stitched and placed on a continuous course of antibiotics for 3 days. The specific surgical procedures are as follows: The bilateral femoral skin and soft tissue were routinely disinfected and shaved, and a longitudinal incision was made at the posterior lateral femur to bluntly separate the subcutaneous tissue and muscle layer, fully exposing the femoral intercondylar fossa. A standardized implant bed was carefully prepared at the predetermined position of the femoral intercondylar fossa using a sterile dental bur under continuous low-temperature saline irrigation to avoid thermal damage to the surrounding bone tissue. After thorough cleaning of the bone cavity and hemostasis, the prepared implant was precisely implanted into the femoral implant bed in place. Subsequently, the muscular layer, subcutaneous tissue and skin were sutured layer by layer with sterile surgical sutures to ensure tight incision closure. All rats received continuous and routine antibiotic prophylaxis for 3 consecutive days after surgery to prevent postoperative infection.

The A-group rats received intragastric administration with 10 mM alogliptin (MedChemExpress, Monmouth Junction, NJ, USA, the purity is 99.92%) five days after surgery. The M-group rats received intragastric administration with 10 mM metformin (MedChemExpress, USA, the purity is 99.64%). The T-group rats received intragastric administration with equivalent saline. Regarding the second animal experiment, the GelMA-group rats received the local injection of the blank hydrogel into the implant bed before implanting implants, while the GelMA + A-group rats received the alogliptin hydrogel delivery system, the quantity of gel used is 10 μL (1%) into the implant bed. All animals were euthanized (euthanasia by overdose anesthesia was performed by intraperitoneal injection of 3% sodium pentobarbital in experimental rats (150–200 mg/kg)) in the 8th week, and the femurs with the implant were harvested for evaluation.

#### 4.3.3. Microscopic, Computerized Tomography (Micro-CT) Assay

The femora with implants were scanned by a micro-CT system (FineTec, Xi’an, Shaanxi, China (Facility location)) and reconstructed with the microtomographic slices. micro-CT scanning was performed with the following parameters: acceleration voltage of 80 kV, current of 70 μA, projection number of 1440, spatial resolution of 10.425 μm, and integration time of 500 ms. VG Studio MAX 3.5 software (VGStudio MAX 3.5 (Version 3.5.0), Volume Graphics GmbH, Heidelberg, Germany)was used for subsequent three-dimensional reconstruction, morphological observation and quantitative analysis of bone–implant integration.

The region of interest (ROI) was formed as an annular domain of 100 μm of bone tissue around the implant. Bone volume per tissue volume (BV/TV), trabecular number (Tb. N) and Tb. The trabecular thickness was estimated to evaluate the osseointegration.

#### 4.3.4. Alizarin Red Bone Labeling

Alizarin Red (30 mg/kg, Sigma, USA) was intraperitoneally injected 10 days before euthanasia to assess new bone formation. After sacrifice, the specimens were cut into 50-μm sections (Finesse Microtome, Thermo Fisher Scientific, Waltham, MA, USA) for histomorphometry. The specimens were visualized by fluorescence microscopy (Axio Imager M; Carl Zeiss, Oberkochen, Germany).

#### 4.3.5. Histology Processing

**Hard Tissue Slicing:** After the completion of micro-CT scanning, the femoral specimens of implants in each group were dehydrated, leached, and embedded in an embedding solution. The longitudinal section parallel to the long axis of the femur was selected as the desired section surface. The tissue-embedded block was fixed in the fixed clip of the hard tissue microtome and sections were performed along the set section direction, ensuring that the thickness of each section was about 250–300 μm to prevent the implant from falling off due to the thin section. The thickness of each section was measured, and the sections of each group were individually glued onto resin slides.

**Paraffin Section and Staining:** Slicing of undecalcified bones was paraffin-embedded, then silicide into 10 μm, and stained for hematoxylin-eosin (HE), modified Masson, Safranin O/Fast Green, Van Gieson (VG), and Toluidine Blue staining.

Immunofluorescence staining for GLP1R (catalog number: ab218532, Cambridge, UK), β-catenin (catalog number: ab32572, Cambridge, UK), and GSK-3β (catalog number: ab32391, Cambridge, UK) was performed to infer the role played by Wnt/β-catenin signaling proteins in osseointegration from the location where they were expressed. Sections were permeabilized in 0.1% Triton X-100 for 20 min and sealed in 10% normal goat serum for 1 h at 27 °C. After draining the blocking solution, different 1/100 antibodies were added to different samples and incubated overnight at 4 °C. After washing three times with PBS, 1/250 Goat Anti-Rabbit IgG (catalog number: ab6721, Cambridge, UK) was incubated for 1 h at room temperature in the dark, then stained with 4′,6-diamino-2-phenylindole (DAPI, Polysciences Inc., Warrington, PA, USA) for 5 min. Immunofluorescence was detected using an inverted fluorescence microscope (Olympus, Tokyo, Japan) after blocking. Immunohistochemical staining was performed to assess the different osteogenic abilities.

Immunohistochemical staining targeting RUNX2 (catalog number: ab92336, Cambridge, UK), BMP2 (catalog number: ab214821, Cambridge, UK) and ALP (catalog number: ab124964, Cambridge, UK) was conducted to quantify osteogenic protein expression around implants. Paraffin sections were deparaffinized and subjected to antigen retrieval with citrate buffer at 95 °C for 20 min. After cooling to room temperature, slides were permeabilized with 0.1% Triton X-100 for 15 min and blocked with 10% normal goat serum for 1 h at 27 °C to eliminate non-specific binding. Following removal of blocking buffer, primary antibodies diluted at 1:100 were applied to each section and incubated overnight at 4 °C. After three PBS washes, 1:200 HRP-conjugated Goat Anti-Rabbit IgG (catalog number: ab6721, Cambridge, UK) was incubated for 1 h at room temperature. DAB chromogenic reagent was used for color development, and hematoxylin was applied for nuclear counterstaining. Images were captured under an upright light microscope (Olympus) for subsequent quantitative analysis of osteogenic staining intensity.

VG staining: Amounts of 9 mL VG staining solution B and 1 mL VG staining solution A were mixed. VG dyeing solution was stained for 3 min, then washed with water and dehydrated with absolute ethanol fast. Finally, transparencies were prepared with clean xylene for 5 min before being sealed with neutral resin.

Toluidine Blue staining: The sections were soaked with the Toluidine Blue dyeing solution for 5 min, rinsed with water, observed continuously to control the depth of staining, and quickly dehydrated and made transparent with absolute ethanol and xylene. Transparencies were prepared with clean xylene for 5 min before they were sealed with neutral resin.

### 4.4. In Vitro Study

#### 4.4.1. Cell Culture

BMSCs and bone marrow-derived mesenchymal macrophages (BMMs) were isolated from the bone marrow of SD rats (approved by the Ethical Committee of the School of Stomatology, The Fourth Military Medical University, Xi’an, China). BMSCs were cultured in Gibco RPMI 1640 complete medium (10% fetal bovine serum, Gibco, Carlsbad, CA, USA), 1% antibiotic solution (penicillin and streptomycin) and incubated in a humidified atmosphere (5% CO_2_, 37 °C). BMMS were cultured in DMEM (high glucose) with 10% FBS and were successively co-cultured with macrophage colony-stimulating factor 1 (M-CSF, 10 ng/mL, ThermoFisher, USA) and lipopolysaccharide (LPS, 200 ng/mL, MedChemExpress, USA) to induce an inflamed type (M1-BMMS) for the test of anti-inflammation properties. The human umbilical vein endothelial cells (HUVECs) were bought from Life Technologies (C0035C, Gibco, USA) and were cultured in Medium 200 (Gibco (Thermo Fisher Scientific), Grand Island, NY, USA) with 2% Low Serum Growth Supplement (LSGS, Gibco, USA) in a humidified atmosphere (5% CO_2_, 37 °C).

#### 4.4.2. Characterization of BMSCs, BMMs, and HUVECs

Flow cytometry was performed to characterize BMSCs based on specific surface antigens. BMSCs were harvested and incubated with relative solutions of antibodies, including *CD29* (catalog number: 4706S), *CD34* (catalog number: 26233), and *CD90* (catalog number: 13801) (Cell Signaling, Massachusetts, CA, USA) according to the manufacturer’s protocols. BMMs were identified by *CD11b* (catalog number: 93169) (Cell Signaling, Massachusetts, CA, USA). HUVECs were identified by morphology and *VE-cadherin*. Data were examined by flow cytometry data analysis software (FlowJo V10, Flowjo, LLC, Ashland, OR, USA).

#### 4.4.3. Cell Viability

Cell counting kit-8 (CCK-8) was used to detect suitable drug concentration and proliferation after alogliptin was used. Cells were resuspended in a 96-well plate and incubated in a medium with different drug concentrations. Afterwards, cells were detected by CCK-8 after 100 nM alogliptin and 200 μM metformin. The optical density (OD) value was analyzed at 450 nm by a microplate reader. The OD of each group was detected by a microplate reader, and the cell proliferation and viability changes under four different drug concentrations were statistically analyzed. Finally, the 100 nM alogliptin and 200 μM metformin with reliable biological activity and no significant cytotoxicity was confirmed for the subsequent animal intervention experiments.

#### 4.4.4. ELISA for BMSCs

For the analysis of VEGF (PV960, Beyotime Biotechnology, Shanghai, China), TNF-α (PT516, Beyotime, China) and IL-10 (PI615, Beyotime, China), ELISA was conducted by supernatants of bone marrow-derived mesenchymal stem cells (BMSCs) after co-culture with saline, alogliptin and metformin upon a 48 h intervention.

#### 4.4.5. Adhesion and Osteogenic Differentiation of BMSCs

An amount of 2 × 10^5^ cells/cm^2^ were incubated in a 48-well plate after the drug dose. After 24 h, cells were washed and incubated with ki-67 and FITC-Phalloidin (Thermo Fisher) according to the protocols. Images were taken using an inverted fluorescent microscope (Nikon Eclipse Ti, Tokyo, Japan).

An amount of 2 × 10^5^ cells/cm^2^ were plated on a 6-well plate with or without alogliptin. After 3 days, the cells were cultured using an osteogenesis induction medium (10% FBS, 0.001 mM dexamethasone (Sigma-Aldrich, St. Louis, MO, USA), 10 mM β-glycerophosphate (Sigma-Aldrich) and 0.05 mM ascorbate-2-phosphate (Sigma-Aldrich)). The OIM medium was changed every 3 days.

#### 4.4.6. Alkaline Phosphatase (ALP) Activity and Alizarin Red Staining

After incubating for 7 days, cells were fixed in 4% paraformaldehyde (Sigma) for 30 min at room temperature. An alkaline phosphatase kit (Jiancheng, Nanjing, China) was added to the plate to perform ALP staining according to the manufacturer’s protocols. ALP activity was identified by a kit (#A059-2, Jiancheng Biotechnology, Nanjing, China) according to the manufacturer’s recommendations.

After incubating for 21 days, cells were fixed in 4% paraformaldehyde (Sigma) for 30 min at room temperature. Alizarin Red S staining (Abcam, Cambridge, UK) was added to the plate to perform calcium nodule staining and quantified at 562 nm after cetyl pyridinium chloride (Thermo Fisher) dissolution.

#### 4.4.7. Tube Formation Assay of HUVECs

The Matrigel matrix gel was placed in a 4 °C refrigerator overnight to melt the day before the experiment. Before the experiment, the Matrigel was placed in a 48-well plate at 200 μL/well with a pre-chilled pipette tip and placed in the incubator to solidify, achieving complete solidity in about 20 min. HUVECs were grouped into the experimental group (A) and the control group (T). They were put back into the incubator, placed in the incubator and continued to be cultured for 4–6 h. The tube formation was observed under the microscope.

#### 4.4.8. mRNA Extraction and Quantitative Real-Time Polymerase Chain Reaction (qRT-PCR) Assay

For cellular molecular detection, after 14 days of osteogenic differentiation of cells, total cellular RNA was isolated and purified strictly in accordance with the Minimum Information for Publication of Quantitative Real-Time PCR Experiments (MIQE) guidelines. Total RNA was extracted from cell samples using a standard RNA isolation kit, and RNA concentration and purity were determined via a spectrophotometer, with an OD260/280 value ranging from 1.8 to 2.1 considered qualified for subsequent experiments. Qualified total RNA was reversely transcribed into complementary DNA (cDNA). Subsequently, quantitative real-time polymerase chain reaction (qRT-PCR) was performed using TB Green^®^ Premix Ex Taq™ II (Takara, Kusatsu, Japan) to detect the relative expression levels of osteogenic differentiation-related genes and key genes involved in the Wnt/β-catenin signaling pathway. The qRT-PCR amplification was carried out on an Applied Biosystems 7300/7500 Real-Time PCR System equipped with ROX Reference Dye II for signal calibration and background correction. The thermal cycling protocol was set as follows: an initial pre-denaturation step at 95 °C for 95 s, followed by 45 amplification cycles consisting of denaturation at 95 °C for 5 s, annealing and extension at 60 °C for 34 s, and dissociation at 95 °C for 15 s. Each sample was analyzed with three independent biological replicates and three technical replicates to ensure experimental reproducibility and eliminate operational errors. The endogenous housekeeping gene GAPDH was employed as an internal reference for normalization. The relative mRNA expression levels of target genes were finally calculated by the 2 comparative threshold cycle method.

### 4.5. Statistical Analysis

All quantitative experimental data in this study were obtained from no fewer than three independent biological replicates and presented as the mean ± standard deviation (SD). Prior to all statistical analyses, the normality of data distribution and homogeneity of variance were systematically tested to verify the applicability of parametric statistical methods. For multiple-group comparative analyses, including cellular experiments, histological staining quantification, micro-CT bone parameter evaluation, and gene expression detection, one-way analysis of variance (ANOVA) was performed, followed by Tukey’s post-hoc multiple comparison test to identify significant differences between groups. All animal experiments were designed with a predefined sample size calculated via G*Power 3.1 software, with seven rats allocated per group to ensure sufficient statistical power and eliminate sampling bias. All statistical calculations were conducted using GraphPad Prism 9.0 software. A two-tailed *p*-value < 0.05 was defined as the threshold for statistical significance. No valid experimental data were arbitrarily excluded during the analysis process, ensuring the accuracy, reproducibility, and rigor of all experimental results.

## 5. Conclusions

This present study showed that type II diabetes impaired the osteogenesis of BMSCs, contributing to osseointegration. Alogliptin enhanced the osteogenesis–angiogenesis immunomodulation of BMSCs derived from type II diabetes rats through the Wnt/β-catenin pathway. We developed an injectable in situ crosslinkable GelMA hydrogel combined with alogliptin to enhance implant osseointegration capacity in type II diabetic rats, systemic alogliptin administration also effectively promotes implant osseointegration under diabetic conditions. Our findings provide a novel vision of the underlying mechanisms of the diabetes implant and offer new insight to patients with diabetes.

### Ethics Approval and Consent to Participate

This retrospective study was conducted in the Department of Oral Implants at the Third Affiliated Hospital of Air Force Medical University from 2012 to 2022. The study followed the Strengthening the Reporting of Observational Studies in Epidemiology (STROBE) statement and was ethically approved by the Ethics Committee of the Third Affiliated Hospital of Air Force Military Medical University (2021133).

All animal experiments were performed following the Institutional Animal Care and the Use Committee of China guidelines and the ARRIVE guidelines. The experiments were approved by the Ethics Committee of the School of Stomatology, Air Force Medical University (Appl. No. kq-2022-011). The 12–13-week-old male Sprague–Dawley (SD) rats (provided by Dossy (Chengdu Dossy Laboratory Animal Co., Ltd., Chengdu, China)) were randomly divided into cages and housed in a 12 h light/dark cycle at 25 °C and 55% humidity. The experiment started with a regular diet for 2 weeks to acclimatize to the environment, followed by a 4-week insulin resistance induction by changing to a high-fat, high-sucrose diet. Anesthesia was performed by intraperitoneal injection of 1% sodium pentobarbital in experimental rats (150–200 mg/kg). A comfortable living environment was provided to minimize rats’ suffering, ensuring sufficient food and water sources. At the end of the experiment, the rats were euthanized by overdose anesthesia.

## Figures and Tables

**Table 1:** Clinical characteristics of the four patient clusters.

	Metformin	Insulin	GLP-1	DPP4i	*p* Value
Demographics					
Number	45	42	33	18	
Age (years)	52 ± 7	52 ± 8	50 ± 6	59 ± 5	0.97
Gender					0.165
Male	30	22	18	9	
Female	15	20	15	9	
HbA1c	6.67 ± 0.42	6.77 ± 0.72	6.65 ± 0.70	6.7 ± 0.68	0.7
Implant					
Length of implant (cm)	10 ± 1	11 ± 1	10 ± 1	9 ± 1	0.18
Level of implant					0.95
Bone-level implant	35 (78%)	27 (47%)	23 (70%)	12 (67%)	
Soft tissue-level implant	10 (22%)	15 (53%)	10 (30%)	6 (33%)	
Brand of Implant					0.59
Straumann	17 (38%)	24 (57%)	14 (42%)	8 (44%)	
Nobel Biocare	11 (24%)	12 (29%)	13 (39%)	6 (33%)	
Takumi	17 (38%)	6 (14%)	5 (15%)	4 (22%)	

## Data Availability

The original contributions presented in this study are included in the article/Supplementary Materials. Further inquiries can be directed to the corresponding author(s).

## Supplementary Materials

The following supporting information can be downloaded at https://www.mdpi.com/article/10.3390/ijms27156800/s1.
