# Supplementary material for: Alogliptin Enhances Implant Osseointegration in Diabetes Through an Osteogenic–Angiogenic Immunomodulatory Procedure
Source: Int J Mol Sci. 2026 Jul 29;27(15):6800. doi: 10.3390/ijms27156800 (PMC13466342; doi:10.3390/ijms27156800)
Supplement: Supplementary file 1 [file ijms-27-06800-s001.zip › Supplementary Tables.pdf]

Table S1. Clinical characteristics of the four patient clusters. (Note: glucagon-like peptide-1, GLP-1; dipeptidyl peptidase-4 inhibitor, DPP4i)

	Metformin	Insulin	GLP-1	DPP4i	p Value
Demographics					
Number	45	42	33	18	
Age (Years)	52±7	52±8	50±6	59±5	0.97
Gender					0.165
Male	30	22	18	9	
Female	15	20	15	9	
HbA1c	6.67±0.42	6.77±0.72	6.65±0.70	6.7±0.68	0.7
Implant					
Length of Implant(cm)	10±1	11±1	10±1	9±1	0.18
Level of Implant					0.95
Bone-Level Implant	35 (78%)	27 (47%)	23 (70%)	12 (67%)	
Soft Tissue-Level Implant	10 (22%)	15 (53%)	10 (30%)	6 (33%)	
Brand of Implant					0.59
Straumann	17 (38%)	24 (57%)	14 (42%)	8 (44%)	
Nobel Biocare	11 (24%)	12 (29%)	13 (39%)	6 (33%)	
Takumi	17 (38%)	6 (14%)	5 (15%)	4 (22%)	

Table S2. Marginal Bone Loss of the four patient clusters. (Note: a, patients in metformin medication cluster compare with patients in DPP4i medication cluster; b, patients in insulin medication cluster compare with patients in DPP4i medication cluster; c, patients in GLP-1 medication cluster compare with patients in DPP4i medication cluster; *, p<0.05)

	Metformin	Insulin	GLP-1	DPP4i
Mesial Marginal Bone Loss (mm)	1.21 ± 0.10 a*	1.37 ± 0.66 b*	1.18 ± 0.25 c	1.05 ± 0.12
Distal Marginal Bone Loss (mm)	1.27 ± 0.20 a*	1.56 ± 0.80 b*	1.28 ± 0.31 c	1.08 ± 0.13
